# Assessment of average glandular dose in mammography practice of a teaching hospital in Ghana

**DOI:** 10.11604/pamj.2024.47.42.39243

**Published:** 2024-02-02

**Authors:** Kofi Adesi Kyei, Samuel Anim-Sampong, Eugene Nartey Ahulu, William Kwadwo Antwi, Joseph Daniels

**Affiliations:** 1 Department of Radiography, University of Ghana, Korle Bu, Accra, Ghana,; 2 National Radiotherapy Oncology and Nuclear Medicine Centre, Korle Bu Teaching Hospital, Accra, Ghana

**Keywords:** Average glandular dose, breast, exposure mode, mammography

## Abstract

**Introduction:**

above the age of 40, women are advised to begin breast examinations and screenings for early detection of breast cancer. The average glandular dose (AGD) provides dosimetric information about the quantity of radiation received by the mammary glands during mammographic exposures. There is, therefore, the need to analyse the radiation dose received by patients presenting for mammography examinations.

**Methods:**

a retrospective cross-sectional design was carried out on the data of 663 participants, conveniently sampled between the months of July 2021 and June 2022. Paired T-test was used to compare imaging parameters for cranio-caudal (CC), medio-lateral (ML), automatic exposure control (AEC), manual exposure control (MEC), and left and right breast. Pearson´s correlation was used to test for relationship between imaging parameters and AGD.

**Results:**

the mean AGD per exposure was 1.9 ± 0.7 mGy for CC projections and 2.3 ± 1.2 mGy for ML projections. The mean AGD per examination for the study was 4.1 ± 1.4 mGy. A positive correlation was found between AGD per examination and exposure factors (tube loading and tube voltage), compressed breast thickness, and compression force. Patient age had no statistically significant relationship with the AGD per examination.

**Conclusion:**

average glandular dose (AGD) was consistent with other findings in literature studies. It was also observed that MEC yielded lower AGD per exposure values than AEC. There was no significant difference in the mean AGD per exposure for left and right breasts.

## Introduction

Breast imaging can be done using mammography, ultrasonography, magnetic resonance imaging, positron emission tomography, or computed tomography [1]. Mammography is however the most widely used modality for breast imaging because of its ability to detect breast pathologies such as breast cancer in its early stages [2]. It uses low dose X-rays to generate images of the internal structures of the breast although other modalities could be used. The breast glandular tissue has a tissue weighting factor of 0.12 [3] which is suggestive of its sensitivity to radiation.

The dose received by the breast tissue is referred to as the average glandular dose (AGD) [4]. According to Gholamkar *et al*. [5] radiation risk is best shown using the AGD and further defined the AGD as the average dose absorbed by the central part of the breast. The determination of the AGD is affected by factors such as the target or filter combination, the thickness of the breast, the X-ray tube current, exposure time, and the peak voltage used during the radiation exposure [6].

One of the studies in Ghana, [7] established that radiation dose in some radiography units was higher when compared with recommended international standards [6]. However, their research was in relation to the spine and chest regions, without any mention of mammography. A search through the literature revealed that there is no available data on the amount of radiation received by patients reporting for mammographic examination. With the current trends of examination in Ghana, it is very difficult to know whether or not mammography patients are being examined with the acceptable limits of radiation dose, and whether or not effective radiation protection procedures are being implemented during these examinations. The unavailability of documentation of mammography doses suggests that patients reporting for mammography examinations may be subjected to higher doses of radiation. Again, there are quite a number of research works in breast-related examination and its treatment such as radiotherapy [8-10]. A search through the literature reveals that there is no available documentation of the dose used in mammography in Ghana. There is, therefore, the need to provide appropriate documentation of mammography doses in the country. This study was therefore conducted to assess radiation dose received by patients undergoing mammography examination in one of the teaching hospitals in Ghana.

## Methods

**Study design:** a retrospective cross-sectional design was carried out on the data of 663 participants who underwent mammography examinations at a radiology department in one of the teaching hospitals in Ghana.

**Study site/setting:** the study was conducted in the Radiology Department of the National Teaching Hospital in the capital city of Ghana between the period of July, 2021 and June, 2022. In this department, both screening and diagnostic mammography are performed. The hospital was selected for the study because it receives an average daily attendance of about 1,500 patients for which mammography examinations form 1%.

**Participants:** folders of 663 participants were conveniently sampled. The mammography unit works three times a week, with an average of 10 cases each day. Only data of patients who had both breasts exposed were included in the study. Participants who fell into the inclusion criteria formed an average of 5 cases each day.

**Variables:** a sample of 15 participants per week was selected, making 720 for the duration of the study. However, the data retrieved from the storage system was 663 because some films (57) were rejected because of underexposure or overexposure.

**Data sources/measurement:** data was collected manually from the mammography system´s computer by recording them with unto the spreadsheet. Upon collection, the data was entered into SPSS for analysis. Tables and bar graphs were used to describe the data.

**Bias:** only the data of patients who had both breasts exposed were included in the study.

**Study size:** the size of the department influenced the selection of the sample.

**Quantitative variables:** Pearson correlation coefficient was used to test for association between the variables. A paired T-test was used to compare and look for differences between parameters grouped into cranio-caudal (CC) and mediolateral oblique (MLO), and left or right. Paired T-test was used to compare imaging parameters for cranio-caudal (CC), medio-lateral (ML), automatic exposure control (AEC), manual exposure control (MEC), and left and right breast. Pearson´s correlation was used to test for relationship between imaging parameters and AGD. One-sample T-test was used to compare findings with some international standards. All inferential analyses were performed with a p<0.05 as the level of significance.

**Statistical methods:** ethical approval was sought from the ethics and protocol review committee of a higher institution as well as the head of the radiology unit of the study site before data collection. The study was also conducted in accordance with the ethical standards laid down in an appropriate version of the 2000 Declaration of Helsinki as well as the Declaration of Istanbul 2008. Any details that sought to disclose the identity of the subjects under the study were omitted. Access to the data collected was restricted to the researchers.

## Results

The results include demographics, the distribution of tube voltage (kVp), tube loading (mAs), CBT, compression force, exposure mode, AGD per exposure and AGD per examination. There are also comparisons and relationships between the mentioned parameters. Results of the age distribution of the 663 patients ranged from 31 years to 87 years, with a mean of 55.3 ± 9.9 years. The most used tube voltage in most of all the CC and MLO projections in the study was the 28 kVp (N=243, 36.7%) and (N=357, 53.8%) respectively. The tube loading ranged from 27 mAs to 574 mAs for all projections. The mean tube loadings for all the CC and MLO projections were 115.7 ± 44.2 mAs, and 148.3 ±79.9 mAs respectively. Only 13 (2.0 %) of the MLO projections exceeded 410 mAs, while no CC projections were made beyond 410 mAs. The mean compression force for the CC and MLO projections were 179.3 ± 26.2 N, and 179.7 ± 21.7 N respectively. The median values for CC and MLO were 186.6 N and 184.7N respectively. The compression force range of 160.00-189.99 N was used for majority (N= 379, 57.1 %) of the CC projections and MLO projections (N= 381, 57.5 %).

**Figure 1:**
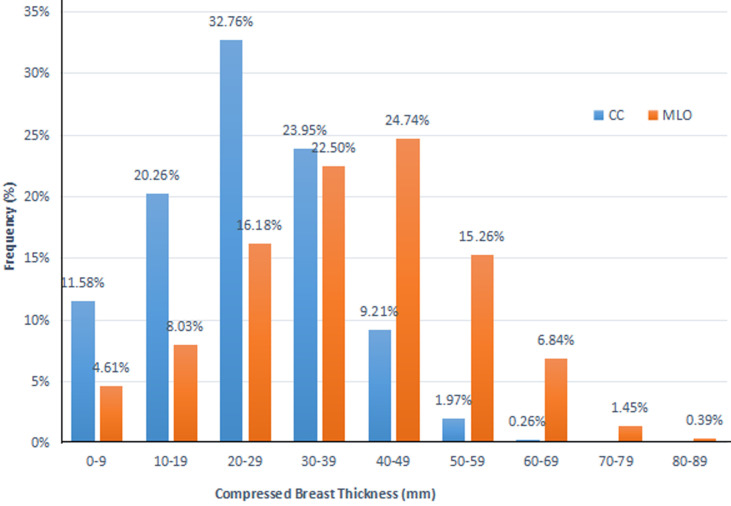
distribution of compressed breast thickness

**Compressed breast thickness (CBT):** the CBT of the patients ranged from 0 mm to 63 mm for CC projections and 0 mm to 82 mm for MLO projections (Figure 1). The mean CBTs were 25.0 ± 12.1 mm for CC and 38.2 ± 15.8 mm for MLO projections. Most (N=218, 32.8%) of the CC projections were performed on the breast in the CBT group 20-29 mm, while more MLO projections were done on the breast in the CBT group 40-49 mm. No CBT exceeded group 60-69 mm for the CC projections.

### Average glandular dose (AGD)

**Figure 2:**
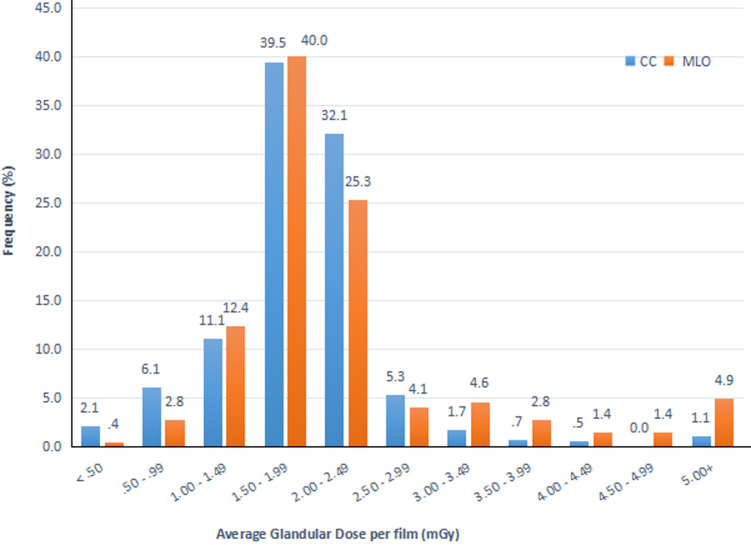
distribution of average glandular dose per exposure

**Average glandular dose per exposure:** in this study, the AGD per exposure for all projections spanned from 0.14 mGy to 8.98 mGy as shown in Figure 2. The mean AGD per exposure during these examinations was 1.9 ± 0.7 mGy for CC projections and 2.3 ± 1.2 mGy for MLO projections. AGD per exposure values in the range of 1.50-1.99 mGy were used for majority of CC (N=262, 39.5%), and MLO (N=265 40%) projections used AGD per exposure values in the 1.50-1.99 mGy group.

**Figure 3:**
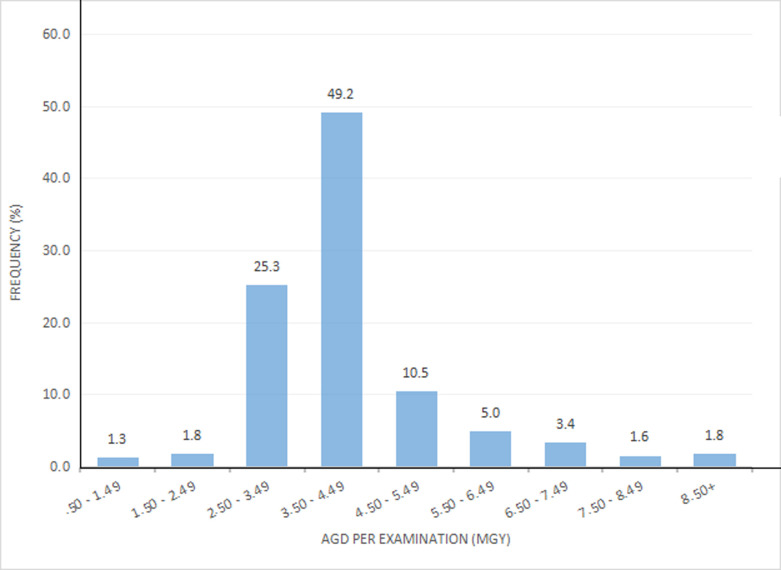
distribution of average glandular dose per examination

**Average glandular dose per examination:** the distribution of AGD per examination is shown in Figure 3. The mean AGD per examination for the study was 4.1 ± 1.4 mGy, with a median of 3.9 mGy. The lowest and highest values were 1.21 mGy and 13.89 mGy respectively. The majority of the participants (N=326, 49.2%) of the patients had AGD per examination values in the range of 3.5-4.5 mGy.

**Distribution of exposure modes and AGD per exposure:** the distributions of the exposure modes and AGD per exposure are displayed in Table 1. All the projections were carried out in MEC and AEC and modes. The AEC exposure mode was used in the majority (N=1095, 72.0%) of all the CC and MLO projections, whereas the manual mode was employed in 425 (28%) cases. The distribution of exposure modes and AGD per exposure showed that 689 (62.9 %) of all the AEC exposures used an AGD per exposure range between 1.50 - 2.49 mGy, while 351 (82.6 %) MEC exposures used the same dose range. Ninety-two (8.4 %) of the exposures made with AEC used doses that exceeded 3.50 mGy whereas 5 (1.2 %) of the exposures made with the manual mode used doses that exceeded 3.50 mGy.

**Table 1:** exposure modes and average glandular dose (AGD) per exposure

AGD per exposure	AEC mode		Manual mode	
	Frequency	Percent, %	Frequency	Percent, %
<0.5	19	1.7	0	0
0.50-1.49	185	16.9	60	14.1
1.50-2.49	689	62.9	351	82.6
2.50-3.49	110	10.0	9	2.1
3.50-4.49	37	3.4	4	0.9
4.50-5.49	23	2.1	1	0.2
5.50-6.49	12	1.1	0	0.0
> 6.5	20	1.8	0	0.0
Total	1095	100.0	425	100.0

Source: field data (2022); AEC: automatic exposure control

From Table 2, it can be seen that there was no statistically significant difference in the CC and MLO compression force (p=0.578). All other parameters were statistically significant. In terms of the exposure mode, AEC recorded higher AGD per exposure and tube loading (mAs) than MEC. MEC on the other hand had higher kVp, CBT, and compression force. The differences in all these parameters proved to be statistically significant as they all had p-values of 0.001.

**Table 2:** comparison of mean values of imaging parameters

Projection		Mean values of imaging parameters			
	Tube voltage (kVp)	Tube loading (mAs)	Compression force (N)	CBT (mm)	AGD per exposure (mGy)
CC	26.6 ± 1.6	115.7 ± 44.2	179.2 ± 24.6	25.0 ± 12.1	1.9 ± 0.7
MLO	27.4 ± 1.5	148.3 ± 79.9	179.7 ± 21.7	38.2 ± 15.8	2.2 ± 1.9
p-value	0.001	0.001	0.578	0.001	0.001
AEC	26.6 ± 1.7	138.8 ± 75.9	177.2 ± 25.4	28.7 ±15.2	2.2 ± 1.1
MEC	28.1 ±0.5	114.4 +24.3	185.2 ± 14.7	39.1 ±13.7	1.8 ± 0.4
p-value	0.001	0.001	0.001	0.001	0.001

Source: field data (2022); CBT: compressed breast thickness; CC: cranio-caudal; MLO: mediolateral oblique; AEC: automatic exposure control; MEC: manual exposure control; AGD: average glandular dose

Table 3 shows that in comparing left and right breasts, the only statistically difference existed for the CBT. All the other parameters had no statistical difference for the breasts. The results showed a positive correlation of R=0.289 and a 0.001 significance between them. Hence, a statistically significant correlation between CBT and compression force was established. Table 4 established a significant negative correlation between patient age and the average CBT for both CC and MLO projections. It also showed that there was no statistically significant correlation (R=-0.05) between the age of the patients in the study and the AGD per examination. The correlation coefficients (R=0.267 - 0.864) and p-values (p=0.001) show significant associations of the above parameters with the AGD per exposure in Table 5.

**Table 3:** comparison of exposure factors for left and right breast

Breast	Mean values of imaging parameters			
	Tube loading (mAs)	CBT (mm)	Compression force (N)	AGD per exposure (mGy)
Left	132.8 ± 78.8	31.3 ± 15.5	179.4 ± 23.8	2.1 ± 1.0
Right	132.7 ± 67.8	31.9 ± 15.6	179.5 ± 22.7	2.1 ± 0.01
p-value	0.978	0.028	0.826	0.992

Source: Field data (2021); R=0.289; p=0.001; CBT: Compressed breast thickness; AGD: average glandular dose

**Table 4:** association between age and average compressed breast thickness for cranio-caudal and mediolateral oblique, average glandular dose per examination

CBT	Age	
	Correlation coefficient	p-value
CC average	-0.116	0.024
MLO average	-0.138	0.007
**Variables**	**Correlation coefficient**	**Significance (2-tailed)**
Patient age	1	0
AGD per examination	-0.05	0.333

Source: field data (2022); CBT: compressed breast thickness; CC: cranio-caudal; MLO: mediolateral oblique; AGD: average glandular dose

**Table 5:** association between compressed breast thickness, compression force, exposure factors, and average glandular dose

Variables	AGD per exposure	
	Correlation coefficient	p-value
CBT	0.413	0.001
Tube voltage	0.267	0.001
Tube loading	0.864	0.001
Compression force	0.143	0.001

Source: field data (2022); CBT: compressed breast thickness; AGD: average glandular dose

## Discussion

Although mammography can be performed for both genders, all the participants of this study (663) were females. This was not a surprising outcome given that breast cancer is more prevalent in females than in males [11] and thus, women are more likely to undergo mammography. The ages of the participants ranged from 31 to 87 years. The minimum age for preparing patients for mammography examination in Ghana is 40 years, unless there is any evidence of possible malignancy where ages below 40 years can be considered. In this study, only 11 (1.6%) participants were below 40 years. The Royal College of Radiologists [12] stipulated that women who presented for breast screening in the United Kingdom were between the ages of 50 to 70 years.

World Health Organisation (WHO) position paper on mammography screening resolved that in well-resourced settings, women aged 50-69 should undergo mammography screening if pre-specified conditions on programme implementation are met [13]. WHO recommends systematic mammography screening in women aged 40-49 years or 70-75 years only in the context of rigorous research and in well-resourced settings. This study utilized data from both screening and diagnostic mammography and thus, a likelihood that some breast malignancies were present in much younger participants. This explains the presence of younger participants in the sample population. A similar study by Jamal *et al*. [14] had 51 years as the median age of the participants whose ages ranged from 31 to 87 years, while Wambani *et al*. [3] reported a mean age of 47 years among a cohort group with age range of 25 to 90 years.

There was a negative relationship between age and CBT for both CC (R = -0.116, p= 0.024) and MLO (R = -0.138, p = 0.007) projections (Table 4). Therefore, patient age increased with decreasing CBT the higher the patient´s age the lower the CBT. A similar study identified a statistical significance (p < 0.001) increase in CBT while adjusting for females age [15]. There was no statistically significant relationship between the participants´ age and AGD per examination. However, the mean of the AGD per examination for participants in the age group 40-49 years was the highest recorded (4.6 mGy) while that of participants in the age group 85-89 years was the lowest (3.1 mGy).

**Average glandular dose per exposure:** the AGD per exposure for all the projections was statistically related to the CBT (R = 0.413, p = 0.001), tube voltage (R = 0.267, p=0.001), tube loading (R = 0.864, p = 0.001) and the compression force (R = 0.143, p < 0.001). The AGD per exposure and CBT for CC and MLO projections, and for left and right breasts using AEC and MEC are presented.

The mean AGD per exposure was 1.9 ± 0.7 mGy for CC projections and 2.2 ± 1.2 mGy for MLO projections. The mean AGD per exposure for CC projections in this study agreed with the recommendation of Sickles *et al*. [16], which states that CC doses should not be more than 3.0 mGy. Both CC and MLO AGD per exposure doses also agreed with the United Kingdom´s recommendation for screening mammography, which states that the AGD per exposure for a standard breast should not exceed 2.5 mGy. The percentage difference in mean AGD per exposure for CC and MLO was 15.9%. The differences between MLO and CC doses were statistically significant with a p-value of 0.001 (Table 4). The reason for the increased dose in MLO projections stems from the fact that MLOs include portions of the pectoral muscles, which causes an increase in the X-ray beam attenuation, requiring more radiation for adequate penetration [17].

From this study, the CBT range for CC and MLO projections were 0 to 63 mm, and 0 to 82 mm respectively. This range was lower than the 10 to 75 mm and 10 to 95 mm for CC and MLO respectively presented by Bouzarjomehri *et al*. [18]. The mean CBT values for this study were 25.0 ± 12.1 mm for CC and 38.2 ± 15.8 mm for MLO. The statistically significant percentage difference between CC and MLO CBTs was 41.7% (p=0.001). Sookpeng *et al*. [17] reported mean CBT of 37.4 ± 14.3 mm and 37.7 ± 16.4 mm for CC and MLO respectively. Generally, the MLO CBT is thicker than the CC but Wambani *et al*. [3] reported that CC and MLO had an equal mean CBT of 40.0 ± 13.0 mm.

Table 3 shows AGD per exposure and the CBT were significantly correlated (R=0.413, p = 0.001). The mean tube loading for CC projections was 115.7 ± 44.2 mAs and that for MLO was 148.29 ± 79.9 mAs. The mean MLO tube loading was 28.19% higher than the mean CC tube loading. The mean compression forces for CC and MLO, 176.2 ± 26.2 N and 179.7 ± 21.7 N respectively, were however not significantly different.

**Comparison of left versus right breast:** the mean CBT for the left breast was 31.25 ± 15.45 mm and that for the right breast was 31.94 ± 15.63 mm, a slight difference. According to Kyei *et al*. [19], the left breast is usually larger than the right breast. The findings of this study proved contrary to Kyei *et al*. assertion, as the mean CBT of the right breast was rather significantly higher (p=0.028) than the mean CBT of the left breast. One reason for this observed difference is that the breasts in this examination were subjected to compression, while Kyei *et al*. [19] described the breast freely hanging. Another factor could be due to differences in the compression force during the examination. Although this study showed that AGD per exposure and CBT are related, this did not lead to any significant differences in doses, as both left and right breasts had mean AGD per exposure values of 2.1 ± 0.1 mGy (p-value=0.992). Only the difference in the left and right CBT was statistically significant. All the other differences between AGD per exposure, tube voltage, tube loading and compression force were not statistically significant.

**Comparison automatic exposure control versus manual exposure control:** Table 2 shows that the mean CBT for MEC and AEC was 39.1 ± 13.7 mm and 28.7 ± 15.2 mm respectively. The mean AGD per exposure for MEC (1.8 ± 0.4 mGy) was however significantly (p = 0.001) lower than that of AEC (2.2 ± 1.1 mGy). This finding was contrary to the report of Harper *et al*. [20], which concluded that AEC doses were higher than MEC doses. The reason for this occurrence was that although the MEC exposures were performed on thicker compressed breasts, there were alterations in the exposure factors. The MEC exposures used a smaller mAs range (50 mAs - 201 mAs) compared with AEC projections (27 mAs - 574 mAs), while the AEC exposures used a smaller mean peak voltage (26.6 kVp) than the MEC exposures (28.1 kVp). However, Table 5 shows that tube loading has a stronger correlation with the AGD per exposure than the tube voltage (R = 0.864 for tube loading, R = 0.267 for tube voltage). Thus, using smaller tube loading values most likely results in the use of a smaller AGD per exposure, hence this observation. Since the MEC exposures were performed on thicker breast, more breast compression was needed to spread out the breast. This resulted in the mean compression force of 185.2 ± 14.7 N for MEC which was significantly higher (p = 0.001) than the mean compression force for AEC exposures, 177.2 ± 25.4 N. According to Table 2, the differences between AEC and MEC in this study were statistically significant for all the parameters tested (tube voltage, tube loading, CBT, compression force, and AGD per exposure) as they all yielded p-values of 0.001.

**Average glandular dose per examination:** from this current mean AGD per examination was 4.1 ± 1.4 mGy and ranged from 1.2 mGy to 13.9 mGy. Table 2 reveals that the AGD per examination in this study was higher than that in the findings of Jamal *et al*. (2003) [14] (3.37 mGy), but lower than that in Bouzarjomehri *et al*. [18] (5.57 mGy), Sookpeng *et al*. [17] (5.96 mGy), and Wambani *et al*. [3] (4.52 mGy). A test for statistical significance yielded p = 0.001 in all instances.

## Conclusion

The mean AGD per examination in this study was 4.1 mGy, while the mean AGD per exposure for CC and MLO were 1.9 mGy and 2.2 mGy respectively. The mean CBT for CC and MLO was 25.0 mm and 38.2 mm respectively. A statistically significant correlation was observed between the AGD per exposure, tube loading, tube voltage, CBT, and compression force (p = 0.001). However, tube loading displayed the strongest correlation with AGD per exposure. The correlation between AGD per examination and age was statistically insignificant (R = -0.05, p = 0.333). Contrarily, this study observed lower values for AGD per examination compared to other studies evaluated with the exception of the findings of other studies where it was higher. It was also observed that MEC yielded lower AGD per exposure values than AEC. There was no significant difference in the mean AGD per exposure for left and right breasts.

### What is known about this topic

- *The determination of the AGD is affected by factors such as the target or filter combination, thickness of the breast;*
- *Radiation risk is best shown using the AGD and further defined the AGD as the average dose absorbed by the central part of the breast;*
- *The breast glandular tissue has a tissue weighting factor of 0.12 which is suggestive of its sensitivity to radiation*.

### What this study adds

- *The study established a significant negative correlation between patient age and the average CBT for both CC and MLO projections;*
- *The study showed a relation between AGD per exposure and CBT with no significant difference in doses identified;*
- *The correlation between AGD per examination and age was statistically insignificant*.
